# Association Between Outdoor Physical Activity and Height Growth Velocity in Chinese Children Aged 9–15: A Secondary Analysis of a National Population-Based Cohort

**DOI:** 10.3390/healthcare14050628

**Published:** 2026-03-02

**Authors:** Yang Yang, Ziyue Sun, Xia Zhong, Jiajia Dang, Shan Cai, Yunfei Liu, Jiaxin Li, Tianyu Huang, Xiaoqian Zhang, Mei Xue, Jing Li, Zhixin Zhang, Yi Song

**Affiliations:** 1 Institute of Child and Adolescent Health, School of Public Health, Peking University, Beijing 100191, China; 2516395133@bjmu.edu.cn (Y.Y.); tyhuang@pku.edu.cn (T.H.); songyi@bjmu.edu.cn (Y.S.); 2 National Health Commission Key Laboratory of Reproductive Health, Beijing 100191, China; 3 Department of Traditional Chinese Medicine, Beijing Children’s Hospital, Capital Medical University, National Centre for Children’s Health, Beijing 100045, China; 4 Graduate School, Beijing University of Chinese Medicine, Beijing 100105, China; 5 Department of Paediatrics, China-Japan Friendship Hospital, Beijing 100029, China; 6 Key Laboratory of Epidemiology of Major Diseases, Ministry of Education, Peking University, Beijing 100191, China; 7 Institute of Clinical Medical Sciences, China-Japan Friendship Hospital, Beijing 100029, China

**Keywords:** linear growth, pubertal growth, sunlight exposure

## Abstract

**Background/Objectives**: The potential of outdoor physical activity as an intervention strategy to promote height growth velocity via stimulating growth hormone secretion and vitamin D synthesis has been scarcely investigated. The present study aimed to investigate the associations between outdoor physical activity duration and height growth velocity, and differences in gender, age, exposure time period (daily, school days vs. weekends), and body mass index (BMI) category. **Methods**: We performed a secondary analysis of longitudinal data from the 2019–2020 Chinese National Survey on Students’ Constitution and Health. The analytic sample included 5029 adolescents aged 9–18 years. High or low height growth velocity was defined as sex- and age-specific percentiles. Associations of high height growth velocity with outdoor activity duration (≥1 h, ≥2 h) on school days and weekends were investigated using multivariable logistic regression models. Analyses were stratified by sex, age group (9–12, 13–15, 16–18 years), and BMI category (normal weight, overweight, and obese). **Results**: Results from this cross-sectional analysis indicate that ≥1 h of daily outdoor physical activity is significantly associated with higher height growth velocity among normal-weight boys aged 9–15 years (OR range: 1.71–2.01) and girls aged 9–12 years (OR = 1.68). The positive association increased with ≥2 h (ORs up to 7.96). Consistently positive associations were found for activity during the school day compared to weekends. No significant associations were found in overweight and obese children. **Conclusions**: Ensuring adequate daily outdoor physical activity—especially on weekends—for at least two hours may be an important potential strategy to promote height growth in normal-weight children and adolescents. Interventions should consider differences in weight status and timing of activity.

## 1. Introduction

Height growth in children and adolescents is an important indicator of their health and nutritional status, which is regulated by multiple factors [1]. Previous studies have shown that height development is mainly regulated by genetic factors (60–80%), but modifiable factors (e.g., lifestyle and nutritional status) also have significant effects on height growth velocity [2]. Outdoor physical activity is an important modifiable factor for height growth, which may promote height growth by two major mechanisms. First, moderate-to-vigorous physical activity stimulates the release of human growth hormone (HGH), thereby promoting the proliferation of epiphyseal cartilage cells [3,4]. Second, exposure to ultraviolet light during outdoor exercise facilitates the synthesis of vitamin D3, which in turn supports/enhances the intestinal absorption of calcium and phosphorus in the intestines, providing the necessary materials for bone mineralization at the growth plate [5,6]. Despite these plausible mechanisms, the majority of existing research has focused on the benefits of outdoor activity in preventing myopia, depression, and overweight and obesity in youth, while its specific role in promoting height growth has received comparatively limited attention [7].

It is worth noting that the biological effects of outdoor physical activity may be most evident during the period of rapid height growth (i.e., before puberty and early adolescence). During puberty, cartilage cells in epiphyseal plates are actively proliferating. The effect of physical activity on growth is most prominent under the synergistic action of HGH and sex hormones. It is crucial to note that sex hormones, especially estrogen, play a complex and central role during this period. While estrogen is associated with the pubertal growth spurt, it is also linked to the eventual closure of growth plates and the cessation of linear growth in both genders. This inherent hormonal complexity during adolescence adds a layer of biological context when examining how extrinsic factors (such as physical activity and nutrition) are associated with height velocity. For instance, the mechanical stress encountered during physical activity correlates with processes like cell division [8,9]. Similarly, adequate vitamin D levels, largely dependent on sunlight exposure, are positively associated with periods of rapid growth. Furthermore, as growth plate closure coincides with late adolescence, the observed relationship between physical activity and height gain also appears to diminish, highlighting the potential importance of developmental timing in understanding these associations [10,11]. Regrettably, despite China’s current “Scientific Fitness Guidelines for Children and Adolescents” recommending at least 60 min of moderate-to-vigorous physical activity daily (preferably outdoors) for ages 6–18, and the “Outline of the National Education Development Plan (2024–2035)” further stipulating no less than 2 h of daily physical activity for primary and secondary students, existing policies lack differentiated outdoor physical activity duration guidelines and evidence-based recommendations tailored to gender and educational stage (e.g., elementary, middle, and high school) [12,13]. However, these guidelines lack evidence-based, differentiated recommendations regarding optimal duration, timing (e.g., school days vs. weekends), or tailored approaches for specific subgroups defined by sex, age, or weight status. Regarding the relationship between overweight and obesity and height, early studies proposed the “nutritional excess-growth promotion” hypothesis [14,15]. However, the abovementioned findings were mostly based on small clinical samples [16]. The potential moderating role of outdoor physical activity in the growth patterns of overweight or obese children and adolescents remains poorly understood.

In order to fill the evidence gaps, we conducted a secondary analysis of the longitudinal data from the 2019–2020 Chinese National Survey on Students’ Constitution and Health (CNSSCH) that used the standardized measurement of height and weight to derive growth velocity and BMI, and self-administered questionnaires to assess the duration of outdoor activity. The specific purposes of this study were (1) to assess the association of outdoor physical activity duration with slower or rapid height growth velocity (according to sex- and age-specific percentiles), separately stratified by sex and age group (9–12, 13–15, and 16–18 years); (2) to check whether a sufficient duration of outdoor physical activity on weekends could offset slower height growth in comparison with shorter duration of outdoor activities on school days; and (3) to assess whether body weight status (normal weight vs. overweight and obese) modifies the observed association between outdoor activity and growth velocity. This study aims to provide detailed evidence on these associations across different contexts and subgroups to inform public health guidelines and interventions.

## 2. Materials and Methods

### 2.1. Study Population

**Figure 1:**
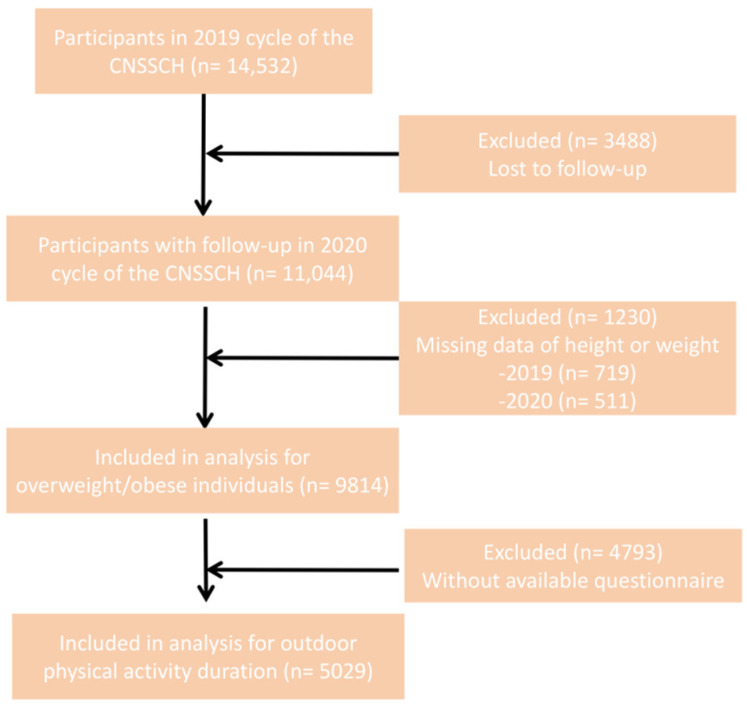
Study flow.

This longitudinal secondary analysis utilized data from the 2019 Chinese National Survey on Students’ Constitution and Health (CNSSCH) [17]. Baseline experimental data were obtained from the 2019 survey wave. A stratified random sampling method was employed, selecting eight provinces representing eastern, central, and western regions of China (Shanghai, Fujian, Shanxi, Henan, Hunan, Gansu, Chongqing, and Guangxi) to ensure geographic and socioeconomic diversity. Follow-up surveys were undertaken in November 2020 within these same provinces. The study involved a 12-month follow-up period from November 2019 to November 2020. A total of 14,532 participants aged 6–18 years completed the baseline survey (Figure 1). After excluding participants aged < 9 years and those with missing data on height or weight, 9814 participants were included in the descriptive analysis. Subsequently, participants with missing key information in the questionnaire (e.g., nutrition-related covariates and self-reported outdoor physical activity) were excluded. The final analytical sample comprised 5029 participants aged 9–18 years (Figure 1). The reduction in sample size was primarily due to missing questionnaire data or inability to reach certain students during the school-based follow-up visit. All procedures followed were in accordance with the ethical standards of the responsible committee on human experimentation and with the Helsinki Declaration. Written informed consent was obtained from all participants and their guardians. The research protocol was approved by the Peking University Institutional Review Board (IRB00001052-18002, IRB00001052-21001) [17]. The study flow diagram was constructed in accordance with the STROBE (Strengthening the Reporting of Observational Studies in Epidemiology) guidelines.

### 2.2. Height Growth Velocity Categorization and Overweight and Obesity Definition

Height and weight were measured by trained technicians using a standard protocol. Height and weight were measured by trained technicians using a portable stadiometer and a digital scale (Model ZDR-P100-ST-01, Nanjing Zhidirui Information Technology Co., Ltd., Nanjing, China) following a standard protocol. Height was measured to an accuracy of 0.1 cm, and weight was 0.1 kg. Height growth velocity was divided into “low” and “high” groups based on whether the annual growth in height was lower or higher than the median of the body height of the overall population of school-age participants. For the purposes of this study, the “elementary school” cohort was defined as 9–12 years, the “middle school” cohort as 13–15 years, and the “high school” cohort as 16–18 years. BMI is defined as weight in kg divided by height in m^2^. The definitions of overweight and obesity are based on gender- and age-specific percentile standards for children and adolescents published by the National Health Commission, with overweight defined as BMI ≥ 85th percentile and obesity defined as BMI ≥ 95th percentile [18].

Rationale for primary categorization method: In the primary analysis, we categorized height growth velocity as “low” or “high” compared with the study population’s median velocity. Since the primary interest was purely for public health attention, this internal (not external) and relative metric is more directly relevant for clinical decision making in individual children and was intuitive and simple to communicate. Our primary classification of child growth simply answers the question: “Is this child growing faster or slower than most of his/her country-mates in the same survey study?” This classification can be useful in detecting those children who are lagging behind the current Caucasian and Asian growth distributions, an issue that a primary concern in school-based health screening. Addressing biological heterogeneity, while we recognize that growth velocity is a function of age, sex, and stage of puberty, we ensured that all downstream regressions were meticulously sex and age stratified (9–12, 13–15, and 16–18 years of age), just as we stratified growth velocity. Sensitivity analysis: In response to the concern about a median split by height velocity (which we did not consider in our primary analysis), we performed a separate pre-planned sensitivity analysis using the continuous and standardized metric. We calculated height-for-age Z-score (HAZ) at baseline and at follow-up for all children using the 2006 Chinese National Growth Standards. Then we used absolute change in HAZ over 1 year (ΔHAZ) as a continuous measure of standardized growth velocity, along with the pre-specified “growth maintenance or acceleration” (ΔHAZ > 0) vs. “growth deceleration or stagnation” (to include children with no growth) (ΔHAZ < 0) as the binary outcome. All primary multivariable models (multilevel logistic and restricted cubic spline analyses) were rerun using ΔHAZ and the binary HAZ-based outcome.

### 2.3. Questionnaire Survey

Self-administered paper questionnaires were given to the school-age participants aged 9–18 years by interviewers [19]. These questionnaires were completed independently by the participants with the interviewer’s assistance. The questionnaire items related to the duration of outdoor physical activity. Other questionnaire sections covered other related variables, including geographic, sociodemographic, and lifestyle variables. The Cronbach’s α of school-related factors in the questionnaire was 0.76, indicating an acceptable internal consistency [20]. The CNSSCH database did not include direct clinical indicators of pubertal development status (e.g., Tanner stages). Accordingly, recognizing the limitation of the data, we stratified all analyses by age and sex as a surrogate for more general developmental status, given the intrinsically tight coupling of pubertal timing to age and sex. This approach cannot take into account the large developmental variance in pubertal maturation among individual offspring within each age–sex stratum.

Outdoor physical activity duration was obtained via student self-administered questionnaires at baseline, with the following question: “In the past week, on average, how much time did you spend daily on outdoor physical activities (e.g., running, ball games, and rope skipping)?” This measurement captured total duration only and did not cover specific activity intensity, type, or context (e.g., physical education class and free play). Furthermore, the questionnaire did not specifically assess indoor physical activity levels. It is important to note that this measurement reflects activity at a single time point (baseline). We assume that reported activity patterns remained relatively stable over the one-year follow-up period, an assumption that may not hold for all participants and constitutes a limitation for longitudinal inference. To acknowledge the methodological limitations, we explicitly note that this self-reported measure of duration cannot differentiate between light, moderate, or vigorous intensity activities. Consequently, the reported “outdoor physical activity duration” serves as a composite exposure metric that may encompass a mixture of intensities and types, and it does not allow for the quantification of moderate-to-vigorous physical activity (MVPA) dose. The inability to assess indoor activity levels also limits our capacity to examine potential substitution effects between indoor and outdoor settings, which could introduce confounding.

### 2.4. Statistical Analysis

Missing data were handled using the hot-deck imputation method [21,22]. This donor-based approach replaces a missing value for a given participant with an observed value from a “donor” participant who is matched on key auxiliary variables (including age, sex, and region). This method preserves the distribution and relationships within the data, reducing bias compared to complete-case analysis. Continuous variables are presented as mean ± standard deviation (SD), and categorical variables as frequencies (percentages). Group comparisons were performed using ANOVA for continuous variables and the χ^2^ test for categorical variables. We used the Mantel–Haenszel test for linear trend to test for trends in proportions for ordered groups.

We used multilevel mixed-effects logistic regression models to examine the association between outdoor physical activity duration and height growth velocity categories. As the data were hierarchically clustered, the joint inclusion of random intercepts for province and school was estimated in each model to take into account the intra-cluster correlation and any unmeasured contextual differences between these levels. Covariates were selected a priori based on the literature and on a directed acyclic graph (DAG) representing the hypothesized causal relationships among the exposure, the outcome, and each of the potential confounding variables. We adjusted for all variables believed to be associated with both exposure and height growth. The fully adjusted model included the following variables, all measured by questionnaire: age as a continuous variable (to account for the physiological growth potential), sex, region of residence (urban/rural as a proxy variable for socioeconomic and environmental circumstances), number of breakfasts consumed weekly, number of occurrences a week of consuming sugar-sweetened beverages, number of eggs and milk per day (to account for the most important nutrients for bone health, including protein, calcium, and vitamin D), and the highest level of parental education (as a measure of socioeconomic status, health literacy, and resource access) [23,24]. We also performed numerous stratified analyses by sex (boys and girls), age group (9–12, 13–15, and 16–18 years), BMI group (normal weight, overweight and obese), activity threshold (≥1 h, ≥2 h), and time period (school days, weekend). Having said that, the reader should be aware that the approach described above leads to multiple comparisons, thereby increasing the risk of Type I error (false-positive findings). To assess the robustness of our findings to alternative operationalization of the outcome, we conducted a set of sensitivity analyses in the logistic regression framework: We ran the full set of multilevel logistic regression models with the outcome “improvement in HAZ” (specified in Section 2.2). To directly examine the concern about dichotomization of the outcome and possible loss of information, we ran an additional analysis where height growth velocity was treated as a continuous outcome. Specifically, we fitted linear mixed-effects models with the same hierarchical structure (random intercept for province and school) and set of covariates as the primary logistic models, with the annual height growth velocity (cm/year) as the continuous dependent variable, and the coefficient of outdoor activity duration as the mean change in growth velocity (cm/year) per unit increment of activity duration. Assessment of the robustness of our results across the models with alternative outcomes, i.e., the logistic model with HAZ improvement and the linear model with continuous growth velocity, is an important step. A robust, significant association across the distinct modeling approaches suggests that our findings are not driven by a particular way of outcome classification or modeling strategy. To examine the possibility of nonlinear dose–response relationships, RCS terms were included in the multilevel models for normal-weight children and adolescents with sex and age group stratification to test and visualize the possibilities of nonlinear dose–response relationships. The inclusion of RCS terms also enhances the numerical interpretability. A three-knot RCS configuration was selected based on comparisons of the Akaike Information Criterion (AIC) and Bayesian Information Criterion (BIC), which indicated it provided the optimal balance of flexibility and parsimony for our sample size. Knots were placed at the 10th, 50th, and 90th percentiles of outdoor activity time to ensure stable estimation across its range. In the overall population model, a formal test for nonlinearity was conducted using a likelihood ratio test comparing the full model (with spline terms) to a reduced linear model. For the continuous variable of outdoor activity time, the odds ratio (OR) was calculated using the median duration in the total sample as the reference (OR = 1). The prediction curves showed how the nadir of the OR for high height growth velocity varied relative to the reference in each stratum. When creating these curves, all other continuous covariates were set at their sample medians, and all categorical covariates, including BMI group, were set at the levels of the reference category, e.g., normal weight for BMI. For sensitivity analyses in the strata where the prevalence of high height growth velocity was >15%, we fit the log-binomial models in the same multilevel structure to obtain prevalence ratios (PRs) to avoid overestimation of odds ratios (ORs). Statistical analyses were performed with the SPSS (version 26.0) and R (version 4.2.2) statistical packages. A two-tailed *p*-value of <0.05 was considered significant. Thus, the exploratory subgroup analyses should be considered with caution.

## 3. Results

### 3.1. Descriptive Results

As shown in Table 1, baseline and follow-up sample sizes remained stable across groups. Urban–rural composition ratios were largely balanced, with only male participants aged 16–18 showing a significantly higher proportion of urban residents (60.9%) compared to rural residents (39.1%).

Regarding body composition indicators, both male and female height growth velocities decreased with increasing educational stage. The average annual height growth for boys aged 9–12, 13–15, and 16–18 years was 8.32 ± 3.61 cm, 5.65 ± 3.76 cm, and 2.79 ± 2.51 cm, respectively; meanwhile, for girls, they were 8.05 ± 4.77 cm, 3.56 ± 2.77 cm, and 2.50 ± 2.42 cm. Boys in the 13–15 and 16–18 age groups showed significantly greater height than girls in the same age groups (*p* < 0.05). Weight gain similarly decreased with increasing educational stage. The average annual weight gain for boys aged 9–12, 13–15, and 16–18 years was 11.21 ± 33.19 kg, 9.48 ± 26.99 kg, and 4.94 ± 14.61 kg, respectively; meanwhile, for girls, they were 8.40 ± 15.59 kg, 4.60 ± 12.61 kg, and 2.86 ± 12.31 kg. Body weight and weight gain were significantly higher in boys than in girls across all age groups (*p* < 0.05). Compared to the elementary-school group, the average BMI values for both boys and girls in the middle-school group increased significantly, while the overweight rate decreased.

**Figure 2:**
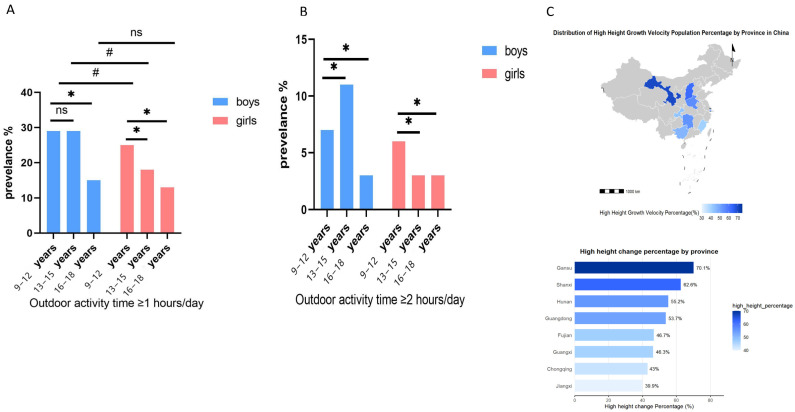
Distribution of outdoor physical activity and its association with height growth among children and adolescents. (**A**) Percentage of boys and girls with daily outdoor physical activity time exceeding 1 h. (**B**) Percentage of boys and girls with daily outdoor physical activity time exceeding 2 h. The symbols in the figure indicate statistical significance: # denotes a significant difference in the qualified rate of daily outdoor physical activity time between genders within the same age group (*p* < 0.05); * indicates a significant difference compared to the 9–12 years age group within each gender (*p* < 0.05); ns indicates no significant difference. (**C**) Percentage of boys and girls with outdoor physical activity time on school days exceeding 2 h. (**C**) Geographic distribution: Percentage of individuals with high height growth in each province.

Regarding average outdoor physical activity time, elementary-school boys showed significantly higher durations than high-school boys, while no significant difference was observed compared to middle-school boys. Elementary-school girls exceeded both middle- and high-school girls. Across all age groups, girls consistently had lower outdoor activity durations than boys (*p* < 0.05, Table 1). The proportion of participants meeting daily activity thresholds (≥1 h or ≥2 h) varied by school stage and gender, with elementary-school students generally showing higher adherence than secondary-school students, and boys consistently exceeding girls. Notably, the high-growth-velocity group demonstrated significantly higher proportions of meeting both activity thresholds compared to the low-growth-velocity group. Geographic variations were also observed, with provinces such as Gansu, Shanxi, and Hunan showing the highest proportions of participants meeting the ≥1 h activity threshold and classified as high growth velocity (see Figure 2 and Figure S1 for detailed values and distributions).

### 3.2. Association Between Daily Outdoor Physical Activity and Height Growth Velocity Classification

**Figure 3:**
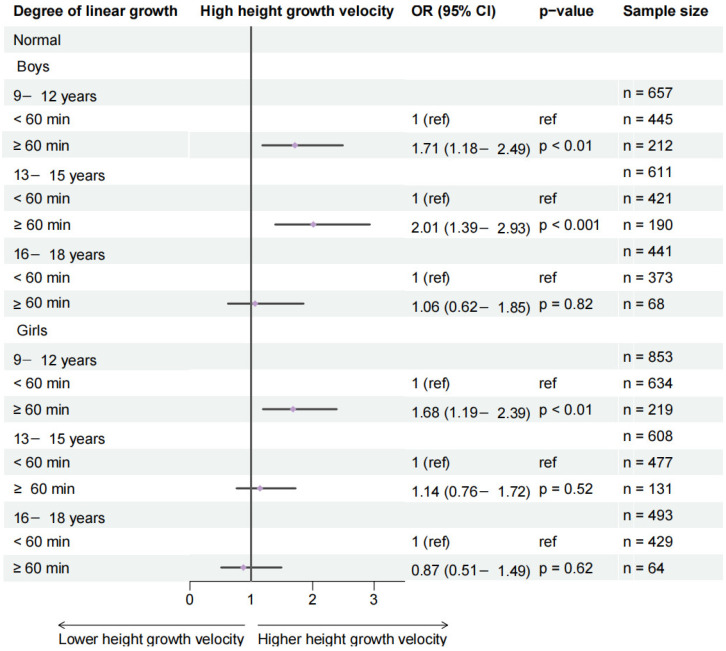
Effects of 1 h of daily outdoor physical activity on changes in height growth velocity categorization in the normal-weight population. Analyses are stratified by age and gender groups and adjusted for area, sugar-sweetened beverage intake, breakfast frequency, daily egg consumption, daily milk consumption, and highest level of parental education.

Among normal-weight children, meeting the threshold of ≥1 h of daily outdoor physical activity was significantly associated with higher height growth velocity in boys aged 9–12 years and 13–15 years, as well as in girls aged 9–12 years. A stronger association was observed for the ≥2 h threshold across all age and sex subgroups, with particularly pronounced effects in younger boys (Figure 3 and Figure S2).

### 3.3. Effects of School Days and Weekend Outdoor Physical Activity Duration on Height Growth Velocity Classification

**Figure 4:**
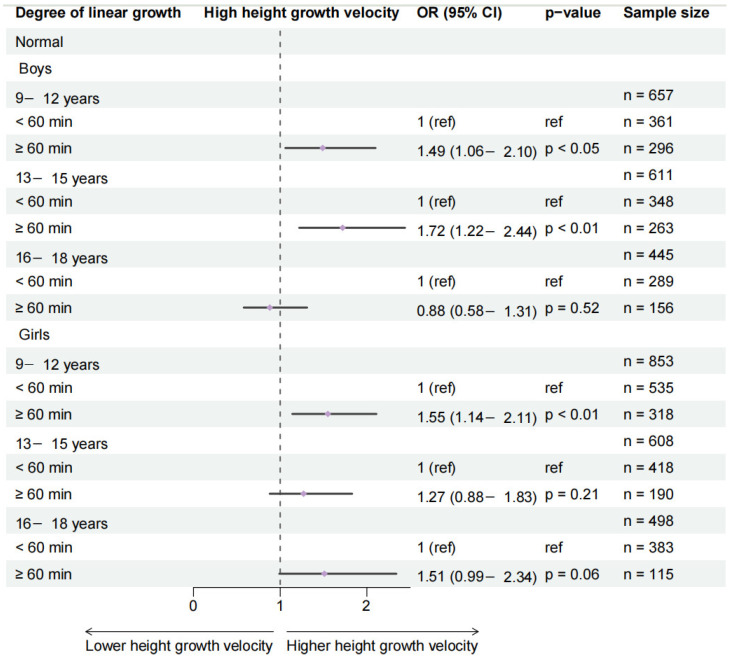
Effects of 1 h of school-day outdoor physical activity on changes in height growth velocity categorization. Analyses are stratified by age and gender groups and adjusted for area, sugar-sweetened beverage intake, breakfast frequency, daily egg consumption, daily milk consumption, and highest level of parental education.

Among normal-weight children, school-day outdoor physical activity (≥1 h) was significantly associated with higher growth velocity only in boys aged 13–15 years and girls aged 9–12 years. The ≥2 h threshold showed broader significant associations across all age and sex subgroups, with particularly strong effects observed in younger boys (Figure 4 and Figure S3).

For weekend outdoor activity, the ≥1 h threshold was significantly associated with higher growth velocity only in girls aged 9–12 years, while the ≥2 h threshold showed significant associations in boys aged 9–12 and 13–15 years, as well as in girls aged 9–12 years (Figure 4 and Figure S3).

### 3.4. Subgroup Analysis by BMI Stratification

**Figure 5:**
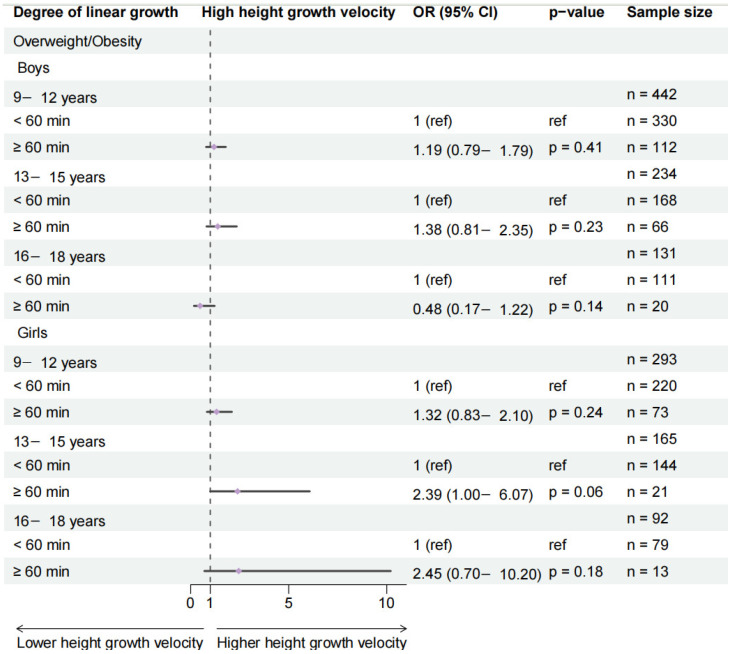
Effects of 1 h of daily outdoor physical activity on changes in height growth velocity categorization among overweight individuals. Analyses are stratified by age and gender groups and adjusted for area, sugar-sweetened beverage intake, breakfast frequency, daily egg consumption, and daily milk consumption. Analyses are stratified by age and gender groups and adjusted for area, sugar-sweetened beverage intake, breakfast frequency, daily egg consumption, daily milk consumption, and highest level of parental education.

Among overweight and obese participants, no significant association was found between school-day outdoor physical activity (≥1 h) and height growth velocity classification across any gender or age group (Figure 5 and Figure S4).

### 3.5. Dose–Response Relationship and RCS Analysis

**Figure 6:**
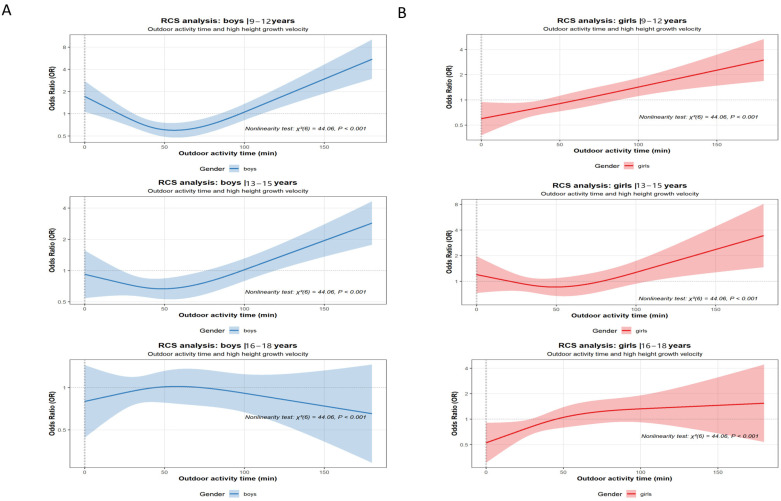
Dose–response relationship between outdoor physical activity duration and the likelihood of positive height growth velocity among children and adolescents with normal weight. (**A**) Logistic regression and RCS curve depicting the dose–response relationship between outdoor physical activity duration and the likelihood of positive height growth velocity among boys with normal weight, analyzed by age group. (**B**) Logistic regression and RCS curve depicting the dose–response relationship between outdoor physical activity duration and the likelihood of positive height growth velocity among girls with normal weight, analyzed by age group.

The RCS analysis suggested potential nonlinear associations between outdoor physical activity duration and the probability of high growth velocity in boys (9–15 years) and girls (13–15 years), with patterns indicative of a U-shaped trend, particularly more pronounced in boys than in girls (Figure 6). However, these curves, especially at the extremes of the activity distribution where confidence intervals widen and data points are sparse, should be interpreted with caution.

### 3.6. Sensitivity Analysis

**Figure 7:**
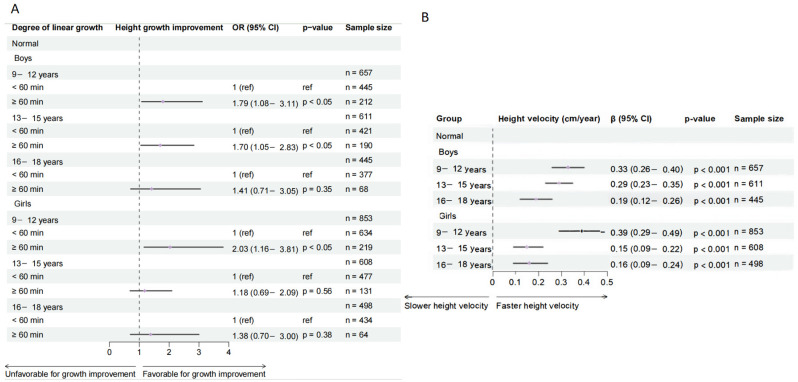
Sensitivity analysis of the association between outdoor physical activity and growth velocity in normal-weight children using AHAZ and linear regression models. (**A**) Results from AHAZ model. (**B**) Results from linear regression model. Analyses are stratified by age and gender groups and adjusted for area, sugar-sweetened beverage intake, breakfast frequency, daily egg consumption, daily milk consumption, and highest level of parental education.

As part of the sensitivity analysis, the results for the normal-weight subgroup, derived using both AHAZ and linear regression models and presented in Figure 7, are consistent with the primary findings reported in Section 3.2. Furthermore, the results for the overweight and obesity subgroups, detailed in Section 5, align with the conclusions drawn in Section 3.4.

## 4. Discussion

Through this multicenter longitudinal study of children and adolescents, we observed significant changes in participants’ height between 2019 and 2020, with the most rapid growth occurring among 9–12-year-old boys and girls. Boys exhibited no significant difference in outdoor physical activity duration between elementary and middle school, but both stages were significantly higher than in high school. In contrast, elementary-school girls had significantly higher activity levels than middle- and high-school girls. The proportion of participants in the high-height-growth-velocity group engaging in ≥1 or ≥2 h of daily outdoor physical activity (including school days and weekends) was significantly higher than in the low-height-growth-velocity group. Daily outdoor physical activity of ≥2 h significantly increased the probability of high growth velocity among 9–15-year-olds, with this association being more pronounced in boys and not evident in overweight and obese individuals.

This study provides the first longitudinal evidence indicating a significant positive association between ≥2 h of daily outdoor physical activity and a higher probability of transitioning from low- to high-growth velocity among 9–15-year-olds. Similarly, ≥2 h of weekend outdoor physical activity was strongly associated with this transition in 9–15-year-old boys and 9–12-year-old girls, whereas no significant association was observed in overweight and obese children. This finding overcomes the limitations of previous cross-sectional studies, providing high-level evidence supporting the role of outdoor physical activity in promoting height growth and informing physical activity policy development for this age group.

Our previous study found that outdoor physical activities affected height growth velocity of preschool children aged 3–6 years, and the effect was especially prominent in 5-year-old girls [25]. Hence, the regulatory function of outdoor physical activities on height might be heterogeneous by gender and age. However, no research has investigated the association between outdoor physical activities and height growth velocity of children and adolescents aged 9–18 years old, and whether the weekend warriors could make up for the reduction of height growth velocity due to insufficient outdoor time on school days [26]. In line with the hypothesis, the present study found that outdoor physical activities played a critical role in the height growth of 9–15-year-olds, and the lack of sufficient outdoor physical activity duration in the school term could be compensated by ≥2 h of outdoor physical activities on weekends to promote the height growth velocity of 9–15-year-old boys and 9–12-year-old girls [27]. The observed associations may be explained by several biologically plausible pathways, though our measurement of outdoor activity duration did not capture intensity. It is well-established in the literature that aerobic exercise of moderate-to-vigorous intensity can stimulate the anterior pituitary gland to secrete HGH, thereby promoting hepatic synthesis of IGF-1, which directly stimulates chondrocyte proliferation [28,29,30,31]. Similarly, activities that involve running or jumping generate mechanical stress, which is known to promote cartilage cell proliferation and local bone metabolism [32,33]. Therefore, to the extent that children’s outdoor activities encompass such moderate-to-vigorous or weight-bearing movements, these pathways could be operative. Moreover, exposure to sunlight during outdoor physical activities can catalyze the conversion of the 7-dehydrocholesterol of skin into vitamin D_3_ under ultraviolet-B radiation. The liver and kidney then metabolize the vitamin D_3_ into the active form, 1,25-(OH)_2_D_3_, which will enhance the intestinal absorption of calcium and increase the bone mineralization [34]. After the ages of 16–18, the increasing sex hormone can accelerate the chondrocyte apoptosis and promote epiphyseal closure, which can reduce the potential of height growth [35,36]. Outside of the generic reference to that, it is important to highlight the specific and central role that estrogen plays in this process. For our population, aged 12–15 years, who are experiencing puberty, estrogen has a dual nature. Estrogen hereby works in synergy with growth hormone to stimulate the pubertal growth spurt, but it is, moreover, the main hormonal signal that terminates linear growth by progressive epiphyseal plate fusion in males and females. This biological internal clock of exponentially increasing estrogen for terminal growth imposes itself as a principal limitation on the hours of window of opportunity for environmental factors such as exercise to affect height. During this period, exercise and vitamin D can only increase the bone density with little effect on height growth.

The observation that reported activity of ≥2 h corresponded to a significantly greater likelihood of accelerated growth versus ≥1 h raises the possibility of mechanistic interpretation, but our findings do not provide direct evidence regarding underlying physiology. If a future study confirms that the reported activity of “outdoor activity for ≥2 h” consistently corresponded to sustained moderate-to-vigorous activity, then the following biologically plausible hypotheses may be considered for the observed association. First, regarding potential hormonal correlates, experimental studies have demonstrated that aerobic exercise can elicit a pulsatile release of HGH, peaking 1–2 h after exercise [37]. It is therefore plausible that a 2 h sustained bout of activity may be associated with a higher magnitude and/or an additional secretory pulse compared to a 1 h period, which could correlate with a greater daily total HGH exposure (a key factor linked to growth plate activity [38]. Second, regarding potential skeletal responses to mechanical load, in line with Wolff’s Law, bone adapts to mechanical loading. It is plausible that increasing the duration of weight-bearing activity, for example, “running and jumping activities”, could be associated with a greater cumulative mechanical strain on the growth plate. This in turn could correlate more strongly with downstream signaling (e.g., ERK1/2) in chondrocytes, and hence with the release of growth-regulatory factors such as osteocalcin, and in turn osteoblast differentiation, compared to a lesser duration of the same activity [39]. Finally, regarding potential links with energy metabolism and nutrition, sustained physical activity can be associated with changes in energy substrate utilization and resulting shifts in appetite. Accordingly, longer activity duration may be linked to a greater anti-catabolic protein environment, when compared to shorter activity durations, and also, perhaps to a greater intake of nutrients relevant to bone growth (e.g., calcium and phosphorus), thereby being correlated with an environment more conducive to bone growth. These are only hypotheses. We did not measure activity intensity, hormone levels, or mechanical load. Accordingly, the observation that the threshold of activity duration corresponded to 2 h rather than 1 h is an important epidemiologic finding that may indicate a critical threshold in the association, the mechanisms of which merit investigation in studies with more precise exposure assessment.

The absence of a significant association between outdoor physical activity duration and the probability of high growth velocity among overweight and obese children and adolescents in our study may be influenced by a confluence of factors, and its interpretation requires balanced caution. First, methodological and statistical considerations offer important non-biological explanations. As noted in the Limitations section, self-reported activity data in this group may be subject to greater measurement error, and for the same reported duration, the actual activity intensity may be lower compared to their normal-weight peers. Furthermore, the reduced sample size in this subgroup after stratification may have limited the statistical power to detect a true but potentially weaker association. Second, at a biological level, existing literature suggests several potential mechanisms. The obese state is often associated with elevated leptin levels and decreased leptin receptor sensitivity, which may perturb hypothalamic regulation of growth hormone release. Obesity-related hyperinsulinemia could suppress hepatic IGF-1 production. Additionally, the sequestration of vitamin D in adipose tissue may contribute to deficiency, potentially affecting calcium metabolism and skeletal health. It is crucial to emphasize that these biological pathways were not directly measured in our study. Therefore, they should be regarded as potential explanatory backgrounds based on prior knowledge, not as definitive causal explanations for our specific findings. In summary, the present results should be interpreted as follows: within the constraints of our study design and measurement methods, no statistically significant association was found between self-reported outdoor activity duration and height growth velocity among overweight and obese adolescents. This does not preclude the possibility of an association being detected with more precise measurements or a larger sample size, nor does it negate the broader health benefits of physical activity for this population. Future research integrating objective measures of activity intensity with relevant biological markers is needed to clarify these relationships.

The highest-priority limitation of this study is that we were not able to adequately adjust for several major determinants of linear growth, and this may lead to residual confounding. First and foremost, we could not adjust for status of pubertal development. Pubertal development is the major determinant of growth velocity in 9–15-year-olds. It is important that readers consider the associations of physical activity and height growth in the context of these major physiological changes. The hormonal changes that come with puberty (e.g., the rise in estrogen) are the key signals that regulate proliferation, maturation, and eventual closure of the epiphyseal growth plates. Thus, individual differences in gender, timing, and tempo of pubertal development are themselves a major determinant of growth velocity and may interact with or confound associations with physical activity. The CNSSCH database did not contain direct measurements of pubertal development (e.g., Tanner stages or hormone levels), and we were not able to adjust for pubertal status as an important covariate in our analyses and include it as a time-varying covariate in our longitudinal models. We stratified all analyses by baseline age and sex categories, and thus we accommodated broadly for pubertal development. However, this approach cannot incorporate the large individual variation in pubertal timing within each stratum. Thus, unmeasured differences in pubertal timing may in fact lead to residual confounding and may have complicated our ability to estimate the association of physical activity independent of these factors. Future studies should consider incorporating biological measurements of pubertal development. Second, the estimates of the association we present (especially the large odds ratios in some subgroups) should be interpreted with some degree of caution as a result of residual confounding due to unmeasured factors and by the sample size. Unmeasured determinants of growth: There was a lack of data on sleep duration and parental height (an important proxy for genetic potential), which could not be incorporated as covariates. The fact that these variables were missing, along with pubertal timing that could not be fully adjusted (first limitation), suggests that residual confounding may exist, either underestimating or overestimating the true magnitude of the associations, and may be partially responsible for the greater magnitude of some estimates of association. In some age–sex strata, the number of participants in the final category of exposure (≥2 h of daily outdoor activity) was relatively small. In observational studies, a lack of sample size in extreme categories of exposure may result in point estimates that are less stable and may inflate the point estimates (i.e., the high odds ratios seen for the younger boys), thereby making them less reliable, and should be interpreted as indicating a strong association rather than the precise magnitude of the odds ratio.

Third, the measurement of physical activity is intrinsically limited in important ways, which directly restrict our mechanistic interpretation of our findings. Time outdoors was assessed by self-report and quantifies only total minutes, providing no information on individual activity types, activity intensity, or context (e.g., within or outside of school physical education). This makes the measure vulnerable to recall and social-desirability bias. It is worth highlighting that the physiological mechanisms we cite here (e.g., stimulation of growth hormone secretion and an effective mechanical load) are typically attributed to moderate-to-vigorous-intensity physical activity (MVPA). Our data do not include any objective assessment of intensity, so we are unable to confirm that the number of minutes of outdoor activity reported by our participants was, at least in part, MVPA. We are therefore unable to establish that the associations we have observed with respect to outdoor activity are driven mechanistically by pathways that can occur only at MVPA intensity. Additionally, our measures did not include any assessment of indoor physical activity. This lack of measurement makes it impossible to adjust for the potential substitution of “underground” activity for “outdoor” activity, which may result in an unmeasured confounder affecting our estimate of the unique effect of outdoor activity. Consequently, we urge caution when interpreting our observed associations in mechanistic terms, given the intrinsic limitation in our measurement of physical activity. We would caution that the associations we report are with the self-reported exposure of minutes of outdoor activity as a composite variable, which plausibly contains mixed intensity, but also other unmeasured exposure. The physiological mechanisms we cited provide a plausible theoretical background but do not directly support our findings. These measurement limitations may be particularly relevant when considering the lack of a significant association in the overweight and obese subgroup of our study. The lack of a significant association between outdoor time and growth velocity in the overweight and obese children/adolescents in our study requires careful consideration from several angles. Firstly, it may reflect some methodological challenges. As above, there may be greater measurement error in the self-reported activity of this group (e.g., differential interpretation of intensity of activity, or social-desirability bias). Additionally, overweight and obese adolescents may participate in activities with a lower actual intensity relative to normal-weight peers for a given value of reported activity duration, an aspect that our data could not address. Second, from a statistical perspective, despite the large overall sample size, the reduction in sample size for the overweight and obese stratum after stratification may have compromised the statistical power to detect a true association which may have been weaker than the true association in the opposite stratum. The size of the sample can also affect the precision and stability of the logistic regression model estimates, which should be considered when interpreting the results for this stratum. Therefore, the lack of association in this stratum must be viewed with caution given these methodological and statistical considerations and should not be interpreted as evidence of no biological effect. Future work will have to rely on an objective multidimensional activity assessment (e.g., accelerometers) that can simultaneously capture activity frequency, duration, and intensity for all BMI categories.

Fourth, the one-year follow-up period is relatively short. It may be too brief to assess the full pubertal growth spurt or long-term growth patterns in the various maturation phases, and it may therefore restrict the generalizability of our results to longer-term growth. Finally, the interpretation of the BMI-stratified results, especially the null result in the overweight and obese subgroup in particular, must be attuned to the possible methodological and statistical issues. Though the lack of a significant association in the high-BMI group is a noteworthy finding, it should be tempered with caution. While we suggested biological mediators (e.g., leptin resistance and vitamin D sequestration) that may explain a weaker effect of physical activity in the context of obesity, we did not directly assess these factors in our analysis, and therefore, these biological mechanisms, while plausible, must be viewed as speculative and alternate, non-biological explanations must be given equal consideration. As we pointed out in the discussion of measurement error in physical activity, misclassification due to greater measurement error in the self-reported data and possibly a lower intensity in activity (for the same self-reported amount of activity) in this stratum could attenuate the true association. In addition, our study measured outdoor activity only at baseline; patterns were assumed to persist across the one-year follow-up period. If activity patterns do indeed change, such exposure misclassification could further attenuate the association. Finally, from a statistical viewpoint, the smaller sample size in the overweight and obese stratum may impact the model’s stability, as well as associated statistical power (i.e., increased risk of Type II error, or false negatives). Second, the large number of stratified analyses performed (by sex, age, BMI, activity thresholds, and time periods) and the multiple comparison problem increase the familywise error rate, i.e., the chance of finding a Type I error (false positives). Our key findings in normal-weight children were robust, but the interpretation of the subgroup results, including the null association in the overweight and obese group, should be made in this context. Thus, the null association should be interpreted as a non-significant association, given the constraints of our study design and measurement, and not as a null biological association. That is to say that a “non-significant association” does not mean that there is no biological association.

## 5. Conclusions

This study’s results have important implications for the design of public health interventions to promote height development in children and adolescents. In line with China’s physical activity guidelines framework, we suggest that policy-making should consider the stage-specific characteristics: schools should implement corresponding policies through the synergistic efforts of families, schools, and communities to ensure that elementary- and middle-school students (especially middle-school girls who previously had less outdoor physical activity time) have at least 2 h of outdoor physical activity time on a daily basis. Taking into account the partial compensatory effect provided by at least 2 h of outdoor physical activity on the weekends, the communities should work together to improve the sports facilities layout (e.g., park green ways and bike lanes dedicated to cyclists) to improve the accessibility and attractiveness of public exercise venues. It is especially important to emphasize that since the height-promoting effect of outdoor physical activity is most evident in normal-weight children, weight control is still an indispensable element in an integrated intervention.

## Figures and Tables

**Table 1:** Demographic characteristics of eligible children and adolescents stratified by age and gender group in the 2019–2020 follow-up study.

	Boy						Girl					
Characteristics	9–12		13–15		16–18		9–12		13–15		16–18	
Years	2019	2020	2019	2020	2019	2020	2019	2020	2019	2020	2019	2020
N	1099	1099	845	845	576	576	1146	1146	773	773	590	590
Age (year)	10.29 ± 1.11	11.34 ± 1.12	13.68 ± 0.89 *	14.71 ± 0.92 *	16.25 ± 0.45 *	17.26 ± 0.45 *	10.21 ± 1.1	11.25 ± 1.13	13.74 ± 0.89 *	14.77 ± 0.94 *	16.22 ± 0.43 *	17.24 ± 0.44 *
Height (cm)	144.95 ± 9.82	153.1 ± 10.58	165.05 ± 8.82 *	170.49 ± 7.4 *	171.59 ± 6.25 *	174.13 ± 6.29 *	144.49 ± 9.07	152.34 ± 8.94	157.55 ± 5.83 #*	160.9 ± 5.78 #*	159.5 ± 5.73 #*	161.79 ± 5.74 #*
Height growth velocity (cm)	8.32 ± 3.61		5.65 ± 3.76 *		2.79 ± 2.51 *		8.05 ± 4.77 #		3.56 ± 2.77 #*		2.50 ± 2.42 #*	
Weight (kg)	39.27 ± 10.93	50.48 ± 36.07	55.08 ± 12.34 *	64.57 ± 24.61 *	62.15 ± 12.12 *	67.09 ± 16.78 *	37.35 ± 9.61 #	45.75 ± 17.74 #	50.79 ± 9.15 #*	55.39 ± 15.06 #*	53.51 ± 9.18 #*	56.36 ± 14.06 #*
Weight change (kg)	11.21 ± 33.19		9.48 ± 26.99 *		4.94 ± 14.61 *		8.4 ± 15.59 #		4.6 ± 12.61 #*		2.86 ± 12.31 #*	
BMI (kg/m^2^)	18.42 ± 3.46	21.15 ± 12.37	20.07 ± 3.46 *	22.09 ± 9.26 *	21.06 ± 3.30 *	22.09 ± 5.2 *	17.68 ± 3.29 #	19.59 ± 7.34 #	20.41 ± 3.22 #*	21.38 ± 5.55 *	21.01 ± 3.23 *	21.51 ± 5.06 #*
Daily outdoor physical activity time (min)	409.13 ± 278.72		452.14 ± 335.10		358.79 ± 245.79 *		379.79± 263.34 #		339.51 ± 240.69 #*		306.57 ± 211.90 #*	
School-day outdoor physical activity time (min)	273.57 ± 214.51		312.33 ± 262.37		251.74 ± 213.37 *		256.32 ± 200.50 #		233.62 ± 196.16 #*		205.74 ± 195.67 #*	
Weekend outdoor physical activity time (min)	135.56 ± 99.75		139.81 ± 104.32		106.64 ± 80.67 *		123.47 ± 95.44 #		105.90 ± 79.22 #*		100.83 ± 82.61 #*	
Area												
Urban	536 (48.8)		417 (49.3)		351 (60.9) *		611 (53.3) #		437 (56.5) #		358 (60.7)	
Rural	563 (51.2)		428 (50.7)		225 (39.1) *		535 (46.7) #		336 (43.5) #		232 (39.3)	
Height growth velocity categorization												
Low	549 (50.0)		418 (49.5)		278 (48.3)		548 (47.8)		358 (46.3)		289 (49)	
High	550 (50.0)		427 (50.5)		298 (51.7)		598 (52.2)		415 (53.7)		301 (51)	
BMI group												
Normal	741 (67.4)	657 (60.0)	633 (74.9) *	611 (72.7) *	462 (80.2) *	441 (77.6) *	915 (79.8) #	853 (74.3) #	615 (79.6) *	608 (78.5) #	495 (83.9) *	493 (83.7) #*
Overweight and obesity	358 (22.6)	442 (40.0)	212 (25.1) *	234 (27.3) *	114 (14.6) *	135 (22.4) *	231 (20.2) #	295 (25.7) #	158 (20.4) *	167 (21.5) #*	95 (16.1) *	97 (16.3) #*

Note: BMI, body mass index. * Comparison between the 9–12 years, 13–15 years, and the 16–18 years age categories (*p* < 0.05). #: denotes a significant difference in the qualified rate of daily outdoor physical activity time between genders within the same age group (*p* < 0.05).

## Data Availability

The data that support the findings of this study are not publicly available due to privacy reasons but are available from the corresponding authors upon request.

## Supplementary Materials

The following supporting information can be downloaded at https://www.mdpi.com/article/10.3390/healthcare14050628/s1, Figure S1: Distribution of outdoor physical activity and its association with height growth among children and adolescents; Figure S2: Association between daily outdoor physical activity duration and changes in height growth velocity among children and adolescents with normal weight, stratified by age and gender; Figure S3: Effects of school-day and weekend outdoor physical activity on changes in height growth velocity among children and adolescents with normal weight; Figure S4: Effects of 1 h of daily outdoor physical activity on changes in height growth velocity categorization among obese individuals; Figure S5: Sensitivity analysis of the association between outdoor physical activity and growth velocity in overweight and obese children using AHAZ.

## Abbreviations

The following abbreviations are used in this manuscript:

BMI	Body mass index
CI	Confidence interval
CNSSCH	Chinese National Survey on Students’ Constitution and Health
HGH	Human growth hormone
IGF-1	Insulin-like growth factor-1
OR	Odds ratio
OWOB	Overweight and obesity
RCS	Restricted cubic spline
