# Supplementary material for: Association Between Outdoor Physical Activity and Height Growth Velocity in Chinese Children Aged 9–15: A Secondary Analysis of a National Population-Based Cohort
Source: Healthcare (Basel). 2026 Mar 2;14(5):628. doi: 10.3390/healthcare14050628 (PMC12984893; doi:10.3390/healthcare14050628)
Supplement: Supplementary file 1 [file healthcare-14-00628-s001.zip › healthcare-4073764-supplementary.pdf]

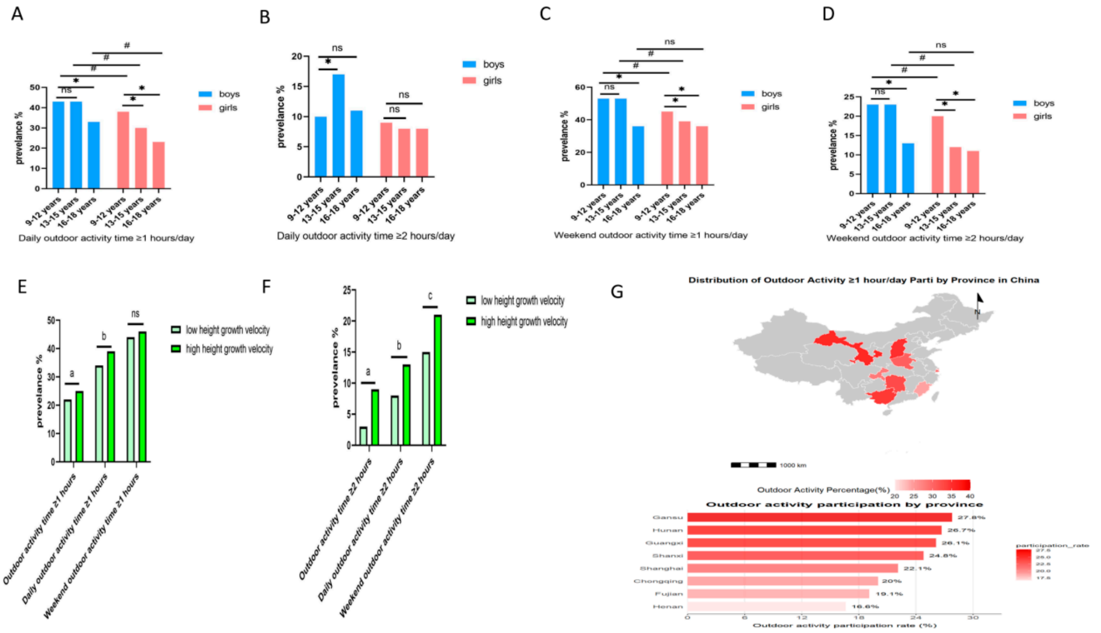

Figure S1. Distribution of outdoor physical activity and its association with height growth among children and adolescents. (A) Percentage of boys and girls with outdoor physical activity time on school days exceeding 1 hour. (B) Percentage of boys and girls with outdoor physical activity time on weekends exceeding 1 hour. (C) Percentage of boys and girls with outdoor physical activity time on school days exceeding 2 hour. (D) Percentage of boys and girls with outdoor physical activity time on weekends exceeding 2 hour.(E) Percentage of individuals with daily, school day, and weekend outdoor physical activity exceeding 1 hour/day, compared between groups with low and high height growth change.(F) Percentage of individuals with daily, school day, and weekend outdoor physical activity exceeding 2 hours/day, compared between groups with low and high height growth change.(G) Geographic distribution: Percentage of individuals with daily outdoor physical activity time exceeding 1 hour/day in each province.

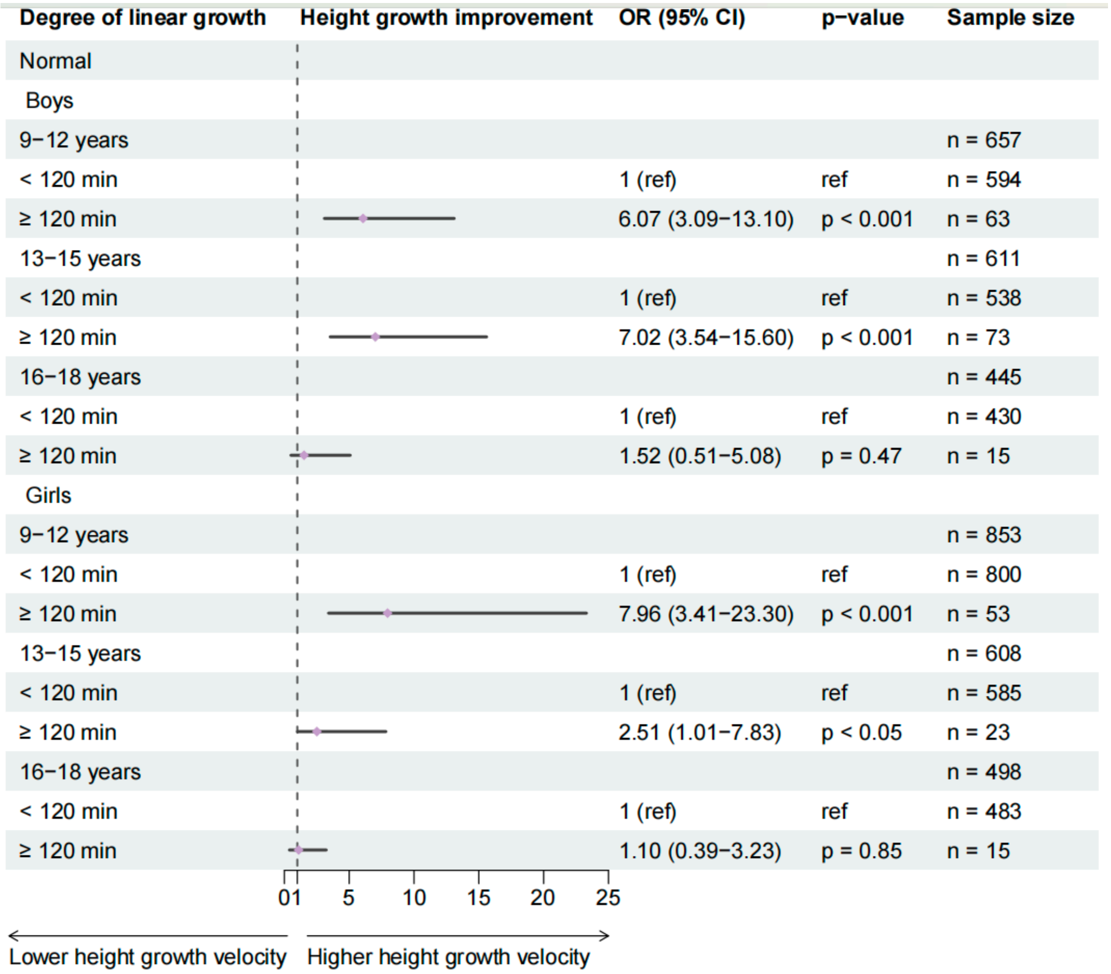

Figure S2. Association between daily outdoor physical activity duration and changes in height growth velocity among children and adolescents with normal weight, stratified by age and gender. Effects of 2 hours of daily outdoor physical activity on changes in height growth velocity categorization in the normal-weight population. Analyses are stratified by age and gender groups and adjusted for area, sugar-sweetened beverage intake, breakfast frequency, daily egg consumption, daily milk consumption, and highest level of parental education.

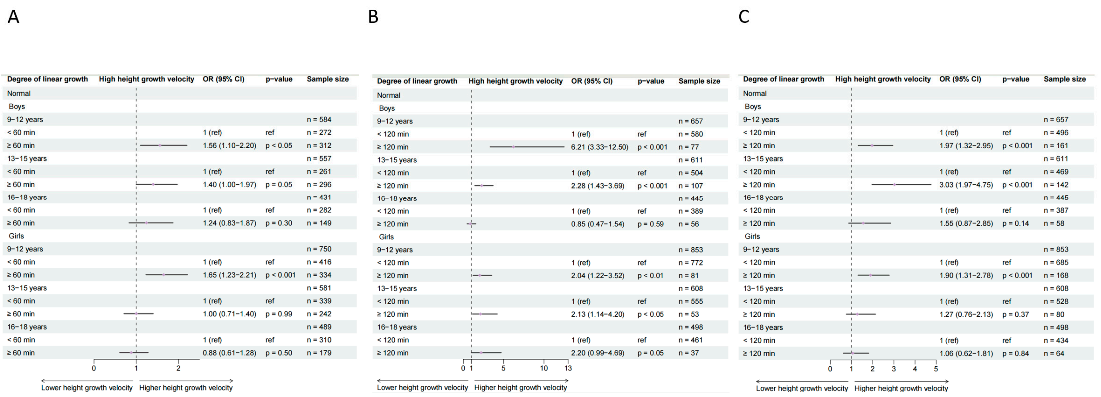

Figure S3. Effects of school-day and weekend outdoor physical activity on changes in height growth velocity among children and adolescents with normal weight. Analyses are stratified by age and gender groups and adjusted for area, sugar-sweetened beverage intake, breakfast

frequency, daily egg consumption, daily milk consumption, and highest level of parental education.(A) Effects of 2 hours of school-day outdoor physical activity on changes in height growth velocity categorization. (B) Effects of 1 hour of weekend outdoor physical activity on changes in height growth velocity categorization.(C) Effects of 2 hours of weekend outdoor physical activity on changes in height growth velocity categorization.

Degree of linear growth	High height growth velocity	OR (95% CI)	p−value	Sample size
Overweight/Obesity				
Boys				
9−12 years				n = 442
< 60 min		1 (ref)	ref	n = 330
≥ 60 min		1.19 (0.79−1.79)	p = 0.41	n = 112
13−15 years				n = 234
< 60 min		1 (ref)	ref	n = 168
≥ 60 min		1.38 (0.81−2.35)	p = 0.23	n = 66
16−18 years				n = 131
< 60 min		1 (ref)	ref	n = 111
≥ 60 min		0.48 (0.17−1.22)	p = 0.14	n = 20
Girls				
9−12 years				n = 293
< 60 min		1 (ref)	ref	n = 220
≥ 60 min		1.32 (0.83−2.10)	p = 0.24	n = 73
13−15 years				n = 165
< 60 min		1 (ref)	ref	n = 144
≥ 60 min		2.39 (1.00−6.07)	p = 0.06	n = 21
16−18 years				n = 92
< 60 min		1 (ref)	ref	n = 79
≥ 60 min		2.45 (0.70−10.20)	p = 0.18	n = 13

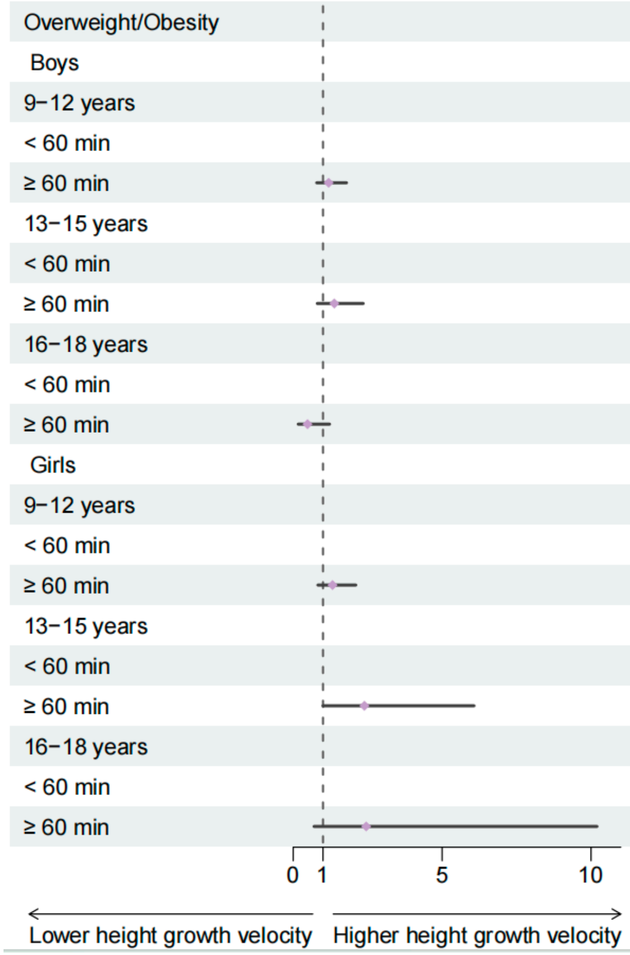

Figure S4. Effects of 1 hours of daily outdoor physical activity on changes in height growth velocity categorization among obese individuals. Analyses are stratified by age and gender groups and adjusted for area, sugar-sweetened beverage intake, breakfast frequency, daily egg consumption, daily milk consumption, and highest level of parental education.

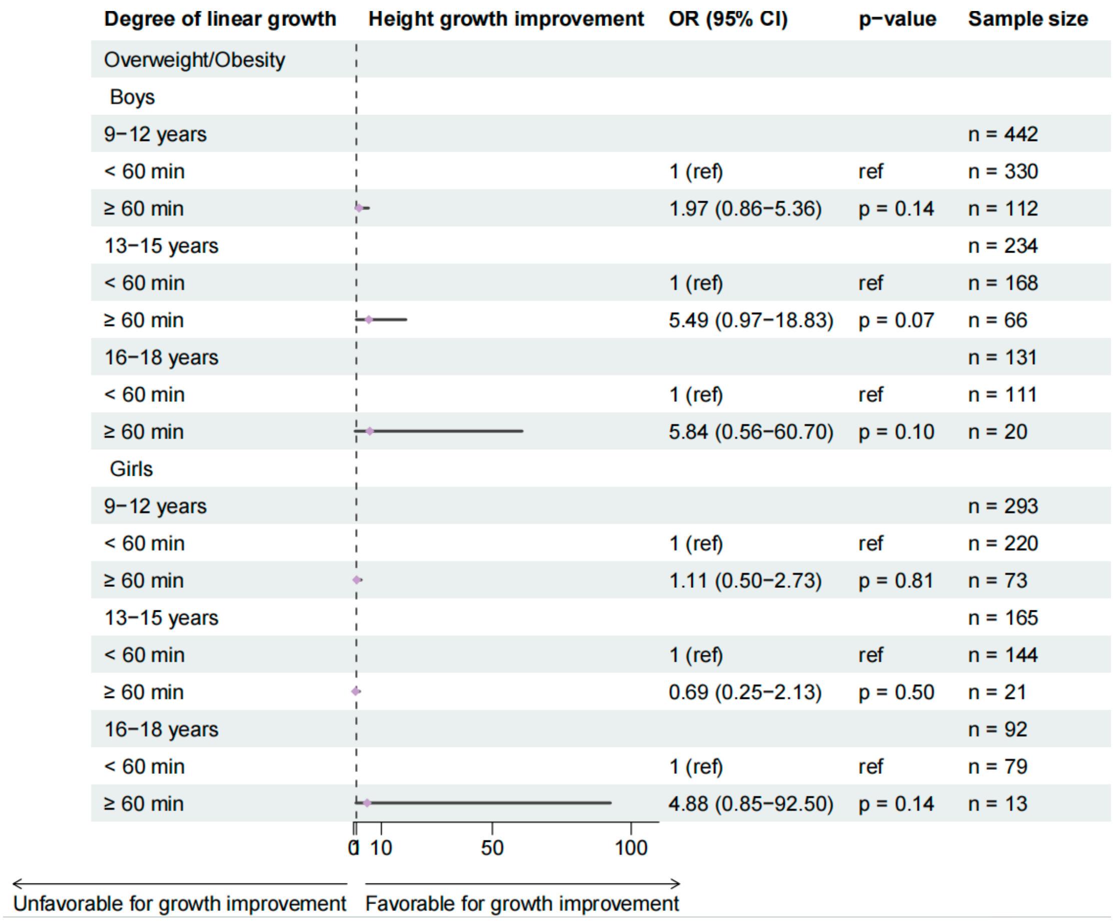

Figure S5. Sensitivity analysis of the association between outdoor physical activity and growth velocity in overweight and obese children using AHAZ .Analyses are stratified by age and gender groups and adjusted for area, sugar-sweetened beverage intake, breakfast frequency, daily egg consumption, daily milk consumption, and highest level of parental education.
